# The supposed tumor suppressor gene *WWOX* is mutated in an early lethal microcephaly syndrome with epilepsy, growth retardation and retinal degeneration

**DOI:** 10.1186/1750-1172-9-12

**Published:** 2014-01-23

**Authors:** Ghada Abdel-Salam, Michaela Thoenes, Hanan H Afifi, Friederike Körber, Daniel Swan, Hanno Jörn Bolz

**Affiliations:** 1 Department of Clinical Genetics, National Research Centre, Cairo, Egypt; 2 Institute of Human Genetics, University Hospital of Cologne, Cologne, Germany; 3 Department of Radiology, University of Cologne, Cologne, Germany; 4 Computational Biology Group, Oxford Gene Technology, Oxford, OX5 1PF, UK; 5 Center for Human Genetics, Bioscientia, Ingelheim, Germany

**Keywords:** *WWOX*, Tumor suppressor gene, Microcephaly, Epilepsy, Retinal degeneration, Nonsense mutation, Whole-exome sequencing

## Abstract

**Background:**

*WWOX*, encoding WW domain-containing oxidoreductase, spans FRA16D, the second most common chromosomal fragile site frequently altered in cancers. It is therefore considered a tumor suppressor gene, but its direct implication in cancerogenesis remains controversial.

**Methods and results:**

By whole-exome sequencing, we identified a homozygous *WWOX* nonsense mutation, p.Arg54*, in a girl from a consanguineous family with a severe syndrome of growth retardation, microcephaly, epileptic seizures, retinopathy and early death, a phenotype highly similar to the abormalities reported in *lde*/*lde* rats with a spontaneous functional null mutation of *Wwox*. As in rats, no tumors were observed in the patient or heterozygous mutation carriers.

**Conclusions:**

Our finding, a homozygous loss-of-function germline mutation in *WWOX* in a patient with a lethal autosomal recessive syndrome, supports an alternative role of *WWOX* and indicates its importance for human viability.

## Background

*WWOX* has been extensively studied for its role in cancer because it localizes to a common fragile site, FRA16D, a genomic region susceptible to chromosomal rearrangements [1]. Indeed, somatic homozygous *WWOX* deletions are common in various malignancies. Early lethality in mice with targeted *Wwox* ablation has largely prevented studies on tumor susceptibility, although focal bone lesions were interpreted as early osteosarcomas. Spontaneous tumor incidence was elevated in heterozygous mice, and exposure to chemical mutagens further promoted tumor formation [2]. Some studies in mice with conditional *Wwox* deletion, however, did not support the categorization of *WWOX* as a primary tumor suppressor gene since no spontaneous neoplasms were observed [3,4]. Recently, expression studies in mice and the phenotype of rats with homozygosity for a spontaneous loss-of-function allele have drawn the attention to a possible role of *WWOX* in the developing central nervous system, including the retina [5-7].

## Methods

### Patients

We have evaluated five members of a consanguineous Egyptian family. Written informed consent was obtained from the parents, and the study was approved by the institutional review board of the Ethics Committee of the University Hospital of Cologne and by the Ethics Committee of the National Research Centre, Cairo.

### Genetic investigations

Genomic DNA of II:4 was subjected to whole-exome sequencing, WES. Exome capture was performed using the Agilent SureSelectXT Human All Exon 50 Mb kit following manufacturer’s procedures (Agilent, Santa Clara, CA, USA) and sequenced with Illumina paired end sequencing (protocol v1.2). Briefly, DNA was sheared by fragmentation (Covaris, Woburn, MA, USA) and purified using Agencourt AMPure XP beads (Beckman Coulter, Fullerton, CA, USA). Resulting fragments were analysed using an Agilent 2100 Bioanalyzer. Fragment ends were repaired and adaptors were ligated to the fragments. The library was purified using Agencourt AMPure XP beads and amplified by PCR before hybridisation with biotinylated RNA baits. Bound genomic DNA was purified with streptavidin coated magnetic Dynabeads (Invitrogen, Carlsbad, CA, USA) and re-amplified to include barcoding tags before pooling for sequencing on an paired-end, 100 cycle run on an Illumina HiSeq 2000 according to manufacturer’s protocols. Exome analysis was completed in OGT’s exome pipeline. Briefly, reads were aligned to the human genome reference sequence GRCh37 using bwa 0.6.2. [8]. Local realignment was performed around indels with the Genome Analysis Toolkit (GATK v1.6) IndelRealigner [9]. Optical and PCR duplicates were marked in BAM files using Picard 1.83 (http://picard.sourceforge.net). Original HiSeq base quality scores were recalibrated using GATK TableRecalibration and variants called with GATK UnifiedGenotyper. Indels and SNPs were hard-filtered according to Broad Institute best-practice guidelines (http://www.broadinstitute.org/gatk/guide/topic?name=best-practices) to eliminate false positive calls. Variant annotation was done with a modified version of ENSEMBL’s Variant Effect Predictor. 14,268,914 read pairs were generated of which 98.96% aligned to the human reference sequence [10]. 85% of sequenced bases were characterized as 'on bait’ or 'near bait’. The sample was sequenced to a mean target coverage of 33.4×, with 64% of bases sequenced to a depth of 20× or more.

## Results

**Figure 1:**
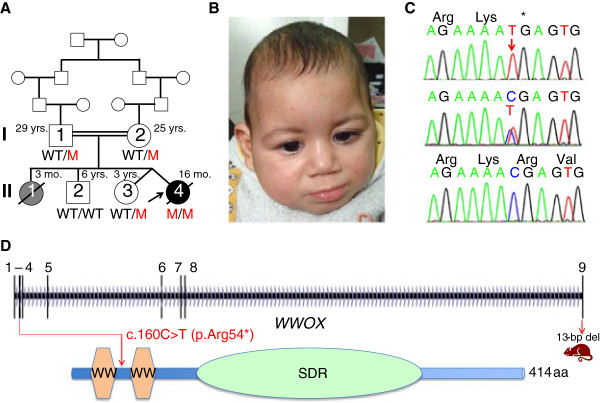
**Clinical and genetic characterization of the consanguineous Egyptian family described herein. A** Pedigree. II:1 is depicted in grey because she likely had the same disorder as II:4 but was never seen by the investigators. M, mutant *WWOX* allele; WT, wildtype *WWOX* allele. **B** Patient II:4 with microcephaly and facial dysmorphism (bitemporal narrowing, high forehead, epicanthic folds, broad base of nose, long philtrum). **C** Sanger sequencing confirmed the homozygous *WWOX* mutation in exon 2, c.160G > T (p.Arg54*) in II:4 (upper panel). Middle: Heterozygosity as seen in both parents and II:3, lower panel: Wildtype sequence in the healthy brother. **D** Scheme of the human *WWOX* gene (vertical bars: exons) and cartoon of the WWOX protein with two WW domains and the short-chain alcohol dehydrogenase/reductase domain. The position of the human mutation (red) and the position corresponding to the rat frameshift mutation (c.1110_1122del/p.Leu371Thrfs*41) are given.

We have evaluated five members of a consanguineous Egyptian family (the healthy parents were 2^nd^-degree cousins) with the index patient, II:4 (Table 1 and Figure 1A,B), affected by a novel syndrome with neonatal growth retardation, microcephaly, retinal dystrophy, severe psychomotor delay and intractable epileptic seizures. She was born at 38 weeks by cesarean section as 2^nd^ twin after an uncomplicated pregnancy. Birth weight of II:4 was 2,000 g (below P3). Birth length and head circumference were not documented, but at 3 months of age weight was 4,200 g (-2.1 SD), length 53 cm (-2.3 SD) and head circumference (OFC) 34 cm (-3.6 SD). At 12 months, her weight was 5,800 g (-3.6 SD), length 66 cm (-2.9 SD) and OFC 39 cm (-4.6 SD). She had bitemporal narrowing, a high forehead, blue sclerae, epicanthic folds, anteverted nares, a long philtrum, a short neck, and clenched hands. Convulsions started at the age of 2 months. At 12 months, seizures started as hemiconvulsions with abnormal eye and mouth movements. Myoclonic movements persisted. Seizures partially responded to valproate and lamotrigine. Deep tendon reflexes of upper and lower extremities and flexor plantar responses were increased. II:4 did not acquire any developmental milestones and never recognized her mother. There were no vocalizations. EEG showed generalized epileptic abnormalities with frequent bursts of sharp and slow waves. Parents noticed lack of response to any visual stimuli at the age of two months, and ophthalmological examination revealed optic atrophy with abnormal retinal pigmentation. Scotopic and photopic flash electroretinogram (ERG) showed moderately reduced response indicating retinal dysfunction. Photopic flicker ERG revealed markedly reduced responses. Visual evoked potential (VEP) showed reduced wave amplitudes and great implicit time delays. In summary, the ophthalmological examination indicated major impairment of retinocortical transmission indicative for delayed visual maturation, and bilateral severe macular and optic nerve dysfunction. Echocardiography and audiological evaluation were normal. Cranial MRI demonstrated supratentorial atrophy with simplified gyral pattern, hypoplasia of the hippocampus and the temporal lobe with consecutively widened subarachnoidal space, and a thin hypoplastic corpus callosum. Chromosomal examination from peripheral blood lymphocytes revealed a normal female karyotype, 46,XX. Normal results were found in metabolic screening including liver and renal function tests, complete blood count, blood gas analyses, ammonia, serum lactate, plasma urea, electrolytes, urine and serum amino and organic acids, and very long chain fatty acids. II:4 died at the age of 16 months because of status epilepticus. No autopsy was performed.

**Table 1:** **Comparison of clinical findings in patient II:4, in the spontaneous rat mutant**[7]**and in knockout mice**[20-22]

	***WWOX***	**MCPH**	**Retinopathy**	**Stature**	**Seizures**	**Brain abnormalities**	**Dev. delay**	**Blood count, metabolism**	**Death**
**II:4**	p.Arg54* homozygous	+	RP, optic atrophy	Short, growth retardation	+	• Supratentorial atrophy	+	• No metabolic disorder	16 mo.
						• Centr./cortical atrophy		• Normal blood count	
						• Hypoplasia of CC		• No tumors	
						• Hippocamp. dysplasia			
						• Temporal lobe hypotrophy			
						• Hippocampal malformation			
						• Gyral pattern anomaly			
**Rat**	p.Leu371Thrfs*41 homozygous (functional null, no protein produced)	n.d.	n.d.	Dwarfism	+	Extracellular vacuoles (hippocampus, amygdala)	+	• Hypogonadism^1^	3–12 weeks
								• No metabolic disorder	
								• Bone metabolic disease (mild)	
								• No tumors	
**Mouse**	Homozygous knockout	n.d.	n.d.	Dwarfism	no	n.d.	+	• Hypogonadism^2^	2–3 weeks
								• Metabolic disorder^3^	
								• Bone metabolic disease (severe)	
								• Osteosarcoma^4^	

^1^Leydig cell dysfunction, apoptosis of spermatocytes, low FSH and LH (prepubertal), low testosterone; ^2^Failure of Leydig cell development; ^3^Hypoproteinemia, hypoglycemia, hypocalcemia, hypotriglyceridemia, hypocholesterolemia, hyponatremia, hyperkalemia, hypochloremia; ^4^Increased susceptibility to develop malignancies was not supported by other studies on *Wwox*-deficient mice (see Discussion).

The 1^st^ twin of II:4, II:3, was a healthy girl. The older sister of II:4, patient II:1, deceased at 3 months of age with similar symptoms, probably affected by the same, presumably recessive disorder. Patient II:1 died several years ago and has not been seen by the authors because the family was only referred to genetic counseling after II:4 had been born with similar symptoms. II:1 was born as first child at 39 weeks of gestation by spontaneous vaginal delivery after an uncomplicated pregnancy. The father and mother were 22 and 26 years old then, respectively. Records on neonatal growth parameters were not available. Seizures started at the age of 40 days as generalized tonic-clonic fits that could partially be controled by valproate and lamotrigine. At 2 months of age, the parents noticed that she was not following objects and did not react to light. No detailed ophthalmological investigations or brain imaging were done. At 3 months, she was admitted to hospital because of heavy convulsions and died one week later during sleep. Autopsy was not performed. There were no additional individuals in the family with symptoms comparable to those of II:1 and II:4. There was no history of cancer, not even in the older generations.

WES identified 41,639 SNPs and indel variant positions, of which 2,597 had not been previously identified in dbSNP release 132. 495 of these were non-synonymous. To reduce the search space for causative mutations, the 41,693 variants were filtered stringently, removing variants that had been seen in OGT’s private database in 30% or more of previously analysed samples. Variants were also removed if they were present in dbSNP release 137 or had been identified as part of the 1000 Genomes project. After this filtering 639 variants (of all consequences) remained. Reducing variants to only exonic regions left 523 novel variants. Assuming that the disease-causing mutation locates in a region of homozygosity by descent (HBD) [11], variants were filtered for homozygous positions. Considering only those variants with a “serious” tag in the OGT pipeline (affecting essential splice sites; frameshifts in coding sequence; gained or lost stop codons; non-synonymous coding variants; complex insertions/deletions) further reduced them to 15 (Table 2). LOH (loss of heterozygosity) regions, probably reflecting HBD segments resulting from parental consanguinity, were identified by analysing the regions surrounding candidate variants in the VCF file, excluding sites with low coverage, known polymorphic genes and potentially mis-genotyped homopolymer indels to define the region sizes presented in Table 2. Only five variants were predicted as deleterious by all or most programs *and* localized to LOH regions, including a gained stop codon in *WWOX* (WW domain containing oxidoreductase) that localizes in exon 2 and affects codon 54 for arginine (c.160G > T; p.Arg54*) in the largest LOH region identified (54 Mb) (chr16:25,457,305-79,894,309; GRCh37) [Additional file 1: Figure S1]. It is homozygous for SNPs with the exception of *PDPR*, a gene exhibiting common copy-number polymorphism. Sanger sequencing confirmed the homozygous *WWOX* mutation in II:4, and both parents and the healthy twin sister (II:3) were heterozygous carriers. The healthy brother (II:2) was homozygous for the wildtype allele (Figure 1C). There are no variants at this position (c.160) of *WWOX* in the Exome Variant Server database. There are some rare missense variants (with minor allele frequencies <1%), but only two other stop gain mutations, c.214C > T (p.Gln72*; rs201008667) and c.749C > G (p.Ser250*; rs368928190). A gained stop codon in *POMC* (proopiomelanocortin) had been sequenced at very low depth (3×) in WES and turned out heterozygous by Sanger sequencing.

**Table 2:** **Apparently homozygous variants in WES data of II:4 remaining after stringent filtering (see Results)**

**Gene**	**Chr**	**Position (hg19)**	**Depth**	**Nucleotide alteration**	**Protein alteration**	**Sift**	**Polyphen**	**Condel**	**Mutation taster**	**Known Mendelian disease gene**	**Cosegreg. with disease**	**LOH region (Mb)**
*POMC*	2	25384251	3	c. 503C > A	p. Ser168*	n.a.	n.a.	n.a.	delet	Early-onset obesity, adrenal insufficiency, red hair; ar	no	no
*IMPG2*	3	100995556	13	c. 535 T > G	p. Ser179Ala (intronic in most transcripts!)	tol	tol	tol	tol	Recessive retinitis, ar	n.d.	20
*PHACTR1*	6	12749777	4	delC	-	n.a.	n.a.	n.a.		no	n.d.	no
*TAS2R38*	7	141672688	34	c. 802C > T	p. Pro268Ser	tol	delet	delet	tol	no	n.d.	2.2
*ERMP1*	9	5832849	3	c. 179C > T	p. Ala60Val	tol	tol	tol	tol	no	n.d.	3
*MRRF*	9	125033286	112	c. 116A > G	p. His39Arg	tol	tol	tol	tol	no	n.d.	2.7
*GLE1*	9	131302562	18	c. 1211C > T	p. Ala404Val	tol	delet	delet	delet	Lethal arthrogryposis with anterior horn cell disease, ar	n.d.	3.7
*HCFC2*	12	104461873	60	c. 461A > T	p. Asn154Ile	delet	delet	delet	delet	no	n.d.	3.3
*RBM19*	12	114261045	18	c. 2867A > C	p. Gln956Pro	delet	tol	tol	tol	no	n.d.	3.6
*SPTBN5*	15	42174192	8	c. 2392_2393ins	p. Glu800Glyfs*41	n.a.	n.a.	n.a.	delet	no; LoF-tolerant gene!	no	no
*DUS2L*	16	68107968	14	c. 842C > G	p. Thr281Ser	tol	tol	tol	delet	no	n.d.	38
*AP1G1*	16	71784192	70	c. 1328A > T	p. Gln443Leu	tol	tol	tol	delet	no	n.d.	1.8
*ZFHX3*	16	72821594	76	c. 10804_10812del	p. Ala3602_Ala3604del	n.a.	n.a.	n.a.	tol	no	n.d.	8.6
***WWOX***	16	78142372	12	c. 160G > T	p. Arg54*	n.a.	n.a.	n.a.	delet	no	yes	54
*ABCG1*	21	43680243	3	c. 718G > A	p. Glu240Lys	tol	tol	tol	tol	no	n.d.	no

Variants in *TAS2R38*, *GLE1*, *HCFC2*, *ZFHX3* and *WWOX* represented prime candidate variants because they localized within an LOH region and were predicted as pathogenic by most programs. Note that the *POMC* nonsense mutation was covered only 3 times, and Sanger sequencing revealed that it was in fact heterozygous which is compatible with *POMC* not localizing in an LOH region. The *SPTBN5* frameshift mutation was found to be homozygous in II:4 and her healthy twin. *SPTBN5* has been described as a non-essential gene that tolerates loss-of-function variants (LoF-tolerant) [15]. ar, autosomal recessive; n.a., not applicable: Assessment of deletions or insertions is out of scope for the methods of the prediction programs which require knowledge of the ancestral amino acids at that position (except Mutation Taster); delet, predicted as deleterious; tol, predicted as being tolerated; n.d., not defined: Segregation analysis was not carried out; LOH region: If a variant is embedded in a run of homozygous SNPs, indicating a loss of heterozygosity region, the extent of this region is given in Mb, megabases.

## Discussion

Many genes have been assigned roles in tumorigenesis at the time of their discovery based on expression studies, somatic alterations in tumor cells or both. For example, *FUS* (fused in sarcoma) was initially classified as a fusion oncogene in human myxoid liposarcomas [12], and later implicated in Mendelian neurodegeneration through the identification of germline mutations in familial amyotrophic lateral sclerosis [13]. Another example is the unexpected identification of germline mutations in members of the Ras signaling pathway in different monogenic syndromes, including a neurodevelopmental disorder, Costello syndrome [14]. Similarly, we have identified a homozygous truncating germline mutation in *WWOX*, a gene so far considered a tumor suppressor gene, in a severe recessive neurodevelopmental syndrome.

**Figure 2:**
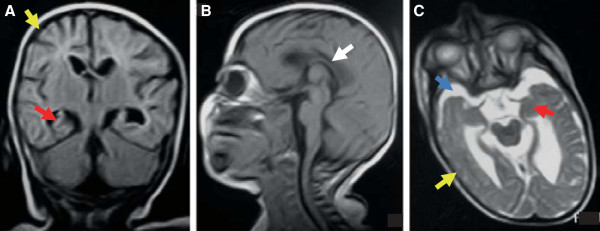
**Brain MRIs of II:4. A** Supratentorial atrophy with thin cerebral cortex (yellow arrow) and hippocampal dysplasia (red arrow) are visible in the coronal plane. **B** Hypoplasia of corpus callosum. Note craniofacial dysproportion and sloping forehead (sagittal MRI). **C** Hypotrophic temporal lobes with widened subarachnoidal space (blue arrow), hippocampal malformation (red arrow) and gyral pattern anomaly (axial MRI).

The implication of *WWOX* in a monogenic disorder may appear unexpected. However, the probability of *WWOX* being a recessive disease gene ranked very high (31) in an estimation for 14,142 genes [15]. This is compatible with only two other stop gain mutations of very low frequency, p.Gln72* (MAF 0.016%) and p.Ser250* (MAF 0.008%), listed in the Exome Variant Server database, likely representing rare pathogenic recessive alleles. Moreover, there is considerable overlap of symptoms in our patients with those observed in *Wwox* murine models (Table 1): A spontaneous homozygous *Wwox* frameshift mutation (p.Leu371Thrfs*41) resulting in non-detectable protein underlies lethal dwarfism and epilepsy (lde) in *lde*/*lde* rats, suggesting an important role of *WWOX* for development and integrity of the nervous system [7]. *lde*/*lde* rats display vacuoles in the hippocampus, a brain region that appeared dysplastic in the MRI of patient II:4 (Figure 2A). Like patient II:4 and *lde*/*lde* rats, knockout mice show growth retardation and die early, but no seizures were observed.

*Wwox* is highly expressed in different brain regions during murine fetal development. Whilst mostly decreasing after birth it remained unchanged in the cortex and the corpus callosum in adult mice [6] – which fits the observation of cortical atrophy and hypoplasia of the corpus callosum in our patients. The intractable epileptic seizures observed in the human family and in the rat model will at least in part result from these anatomical brain abnormalities, but considering the putative role of WWOX as a signaling protein [16], disruption of neuronal pathways may additionally increase the susceptibility for seizures.

Severe retinal degeneration and optic atrophy were present in II:4 and probably in II:1, too, and compatible with early infantile retinitis pigmentosa, RP. Interestingly, *Wwox* is moderately to highly expressed in the murine developing retina [6] and overexpressed in the inner and outer nuclear layers after light-induced damage [5]. It colocalizes with opsin-positive cones and was enriched in damaged mitochondria and condensed nuclei of degenerating photoreceptors [5]. Intriguingly, *Wwox* was upregulated with age in retinae of *rd* mice with mutations in *Pde6b*, a recessive RP gene in humans [17]. In monogenic forms of optic atrophy, the disease primarily affects the retinal ganglion cells (RGCs) [18]. The specific expression of *Wwox* in RGCs, its colocalization with Brn3b [6], a protein that is crucial for RGC axon outgrowth [19], and continued expression in the optic tract in adulthood could explain the optic atrophy in our patients.

By interaction with many proteins via its WW and SDR domains, WWOX is thought to act in various signaling pathways [2,16]. In *WWOX* deficiency, disease in the central nervous system may result from disruption of some of these interactions, e.g. with dishevelled segment polarity protein 2 (DVL2) and Tau, proteins involved in cortical development and neurodegeneration [2]. The severe dwarfism in *Wwox*^-/-^ mice has been attributed to delayed bone formation resulting from dysregulation of RUNX2, a WWOX-controlled regulator of skeletal morphogenesis, and to down-regulation of genes for nucleosome assembly and cell growth [20].

Metabolic disease has been reported in *Wwox*-deficient mice (see legend for Table 1), but was not evident during her short life in patient II:4. Spontaneous tumor incidence was moderately elevated in heterozygous *Wwox* knockout mice compared to wildtype, and exposure to chemical mutagens dramatically promoted tumor formation [21]. However, some mouse studies raised doubts on the gene’s role as a classical tumor suppressor [3,4,20-22], and neither the rat model nor members from the human family reported here displayed a tendency to develop tumors. It is possible that tumors would have developed later in life in II:1 and II:4 if the condition would have been compatible with a longer survival. No tumors have developed so far in the heterozygous carriers in our family, and there was no history for cancer in the extended family (with possibly several carriers of the mutation). However, an increased cumulative lifetime risk, especially if exposed to carcinogenic agents, seems conceivable and should result in regular preventive medical checkups.

## Conclusions

Our findings – albeit based on the presumption that the causative mutation in this consanguineous family will be homozygous (we can not exclude disease-causing compound heterozygosity in a gene outside the HBD regions) – suggest a redefinition of WWOX as a protein that has entirely different functions in the germline versus the soma. It joins the ranks of other tumor suppressor genes that have been recognized to be crucial for neurodevelopment when mutated in the germline.

## Competing interests

HJB is employee of Bioscientia which is part of a publicly traded diagnostic company. The other authors have no competing interests.

## Authors’ contributions

GAS, HHA and FK carried out the clinical characterization of the family. MT carried out the molecular genetic studies apart from exome sequencing. DS performed exome sequencing and bioinformatic/statistical analysis. GAS and HJB designed the study. HJB wrote the manuscript. All authors read and approved the final manuscript.

## Supplementary Material

Additional file 1: Figure S1 Schematic representation of the mapped sequencing reads covering the *WWOX* mutation in patient II:4 (visualized with the Integrative Genomics Viewer, IGV). Click here for file
