# Supplementary figures and images for: The supposed tumor suppressor gene WWOX is mutated in an early lethal microcephaly syndrome with epilepsy, growth retardation and retinal degeneration
Source: Orphanet J Rare Dis. 2014 Jan 23;9:12. doi: 10.1186/1750-1172-9-12 (PMC3918143; doi:10.1186/1750-1172-9-12)

## Slide 1
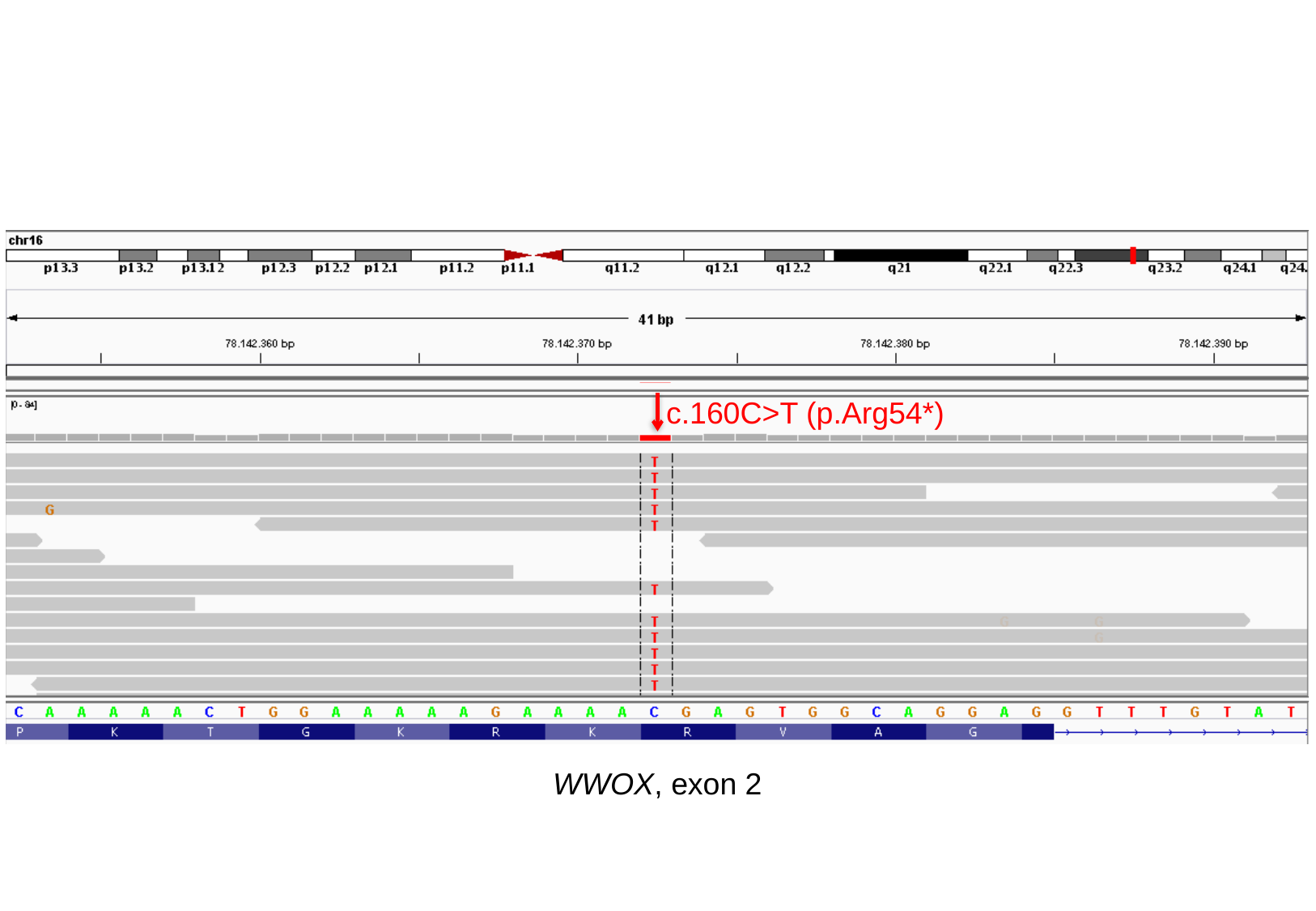

c.160C>T (p.Arg54*)
WWOX, exon 2

Supplement: Additional file 1: Figure S1 — Schematic representation of the mapped sequencing reads covering the WWOX mutation in patient II:4 (visualized with the Integrative Genomics Viewer, IGV). [file 1750-1172-9-12-S1.pptx]
